# Faecal Calprotectin in Assessment of Mucosal Healing in Adults with Inflammatory Bowel Disease: A Meta-Analysis

**DOI:** 10.3390/jcm10102203

**Published:** 2021-05-19

**Authors:** Mariusz A. Bromke, Katarzyna Neubauer, Radosław Kempiński, Małgorzata Krzystek-Korpacka

**Affiliations:** 1 Department of Biochemistry and Immunochemistry, Wroclaw Medical University, Chalubinskiego 10, 50-368 Wroclaw, Poland; malgorzata.krzystek-korpacka@umed.wroc.pl; 2 Department of Gastroenterology and Hepatology, Wroclaw Medical University, Borowska 213, 50-556 Wroclaw, Poland; katarzyna.neubauer@umed.wroc.pl (K.N.); radoslaw.kempinski@umed.wroc.pl (R.K.)

**Keywords:** inflammatory bowel disease, mucosal healing, faecal calprotectin, diagnostics, meta-analysis

## Abstract

Achieving mucosal healing in patients with inflammatory bowel disease is related to a higher incidence of sustained clinical remission and it translates to lower rates of hospitalisation and surgery. The assessment methods of disease activity and response to therapy are limited and mainly rely on colonoscopy. This meta-analysis reviews the effectiveness of using faecal calprotectin as a marker for mucosal healing in inflammatory bowel disease. Two meta-analyses were conducted in parallel. The analysis on the use of faecal calprotectin in monitoring mucosal healing in colonic Crohn’s disease is based on 16 publications (17 studies). The data set for diagnostic values of faecal calprotectin in ulcerative colitis is composed of 35 original publications (total 49 studies). The DOR for the use of faecal calprotectin in Crohn’s disease is estimated to be 11.20 and the area under the sROCis 0.829. In cases of ulcerative colitis, the DOR is 14.48, while the AUC sROC is 0.858. Heterogeneity of the studies was moderatetosubstantial. Collected data show overall good sensitivity and specificity of the faecal calprotectin test, as well as a good DOR. Thus, monitoring of mucosal healing with a non-invasive faecal calprotectin test may represent an attractive option for physicians and patients with inflammatory bowel disease.

## 1. Introduction

Inflammatory bowel disease (IBD) is an umbrella term for ulcerative colitis (UC) and Crohn’s disease (CD), which are lifelong, severe conditions of the gastrointestinal tract, characterized by heterogeneous clinical presentation, a relapsing-remitting course and a wide spectrum of complications including extraintestinal manifestations [1,2,3].

Because of the complex and unclear pathogenesis of IBD, an effective causative treatment strategy of the disease is still missing. This, in turn, intensifies clinical trials not only of novel therapeutic agents, but also on therapeutic goals—especially important, considering the chronic and unpredictable course of IBD. A treat-to-target strategy aims to limit IBD progression and improve outcomes by adjusting therapy according to the achievement of predefined treatment response targets. The STRIDE (Selecting Therapeutic Targets in Inflammatory Bowel Disease) program identified the therapeutic targets for both UC and CD, as clinical/patient-reported outcome (PRO) remission and endoscopic remission. In addition, for CD, the resolution of inflammation-related findings on imaging in patients who cannot be adequately assessed with ileocolonoscopy [4,5]. The concept of deep remission (DR) is complex, and a standardised definition of DR is still missing [6]. Deep remission in UC refers to complete disease quiescence regarding endoscopic activity, rectal bleeding, and bowel movement [7].

Identification of mucosal healing (MH) as a new therapeutic goal, different from clinical remission, has revolutionised the approach to IBD management. One weakness of MH is the need to use endoscopy for its evaluation, which is an invasive, time-consuming, and expensive technique. Moreover, there is no commonly accepted definition of MH, although several scales or indices for objective classification of endoscopic findings have been devised. At the moment, the Mayo endoscopic score (MES) [8,9], is frequently usedin the evaluation of treatment efficacy in clinical trials, with MH defined as MES ≤ 1. However, the guidelines of boththe European Crohn’s and Colitis Organisationand the Japanese Society of Gastroenterologyrestrict complete endoscopic remission to a score of zero (normal or completely healed mucosa) [10,11,12]. The only two indices that received formal validation in UC, are the Ulcerative Colitis Endoscopic Index of Severity (UCEIS) and the Ulcerative Colitis Colonoscopic Index of Severity (UCCIS). In the UCEIS, an index value corresponding to endoscopic remission has not been defined, although MH is most often described as 0–1 point. Endoscopic activity of Crohn’s disease may be reliably scored using either the Crohn’s Disease Endoscopic Index of Severity (CDEIS) [13] or the Simple Endoscopic Score for Crohn’s Disease (SES-CD) [14]. Both scales have been prospectively validated and are highly reproducible, with excellent inter-observer agreement.

Indeed, a catalogue of benefits associated with achieving MH in IBD justifies repeated endoscopic examinations, as it encompasses a more favorable course of the disease and is related to fewer surgeries and hospitalisations, as well as with a long-term clinical remission. For instance, mucosal healing in UC is accompanied by a lower risk of immunosuppression, colectomy, and colitis-associated neoplasia [15,16,17,18]. In turn, MH in CD is related with less severe inflammation after five years, decreased risk of future steroid treatment, and lower rates of surgical resection [5,19,20,21]. Successful control of intestinal inflammation may lead to improvement of extraintestinal manifestations of IBD, such as peripheral arthralgia [22], as well as relief in IBD-concomitant anxiety, depression, fatigue, and sleep disturbances [23]. Therefore, endoscopy, which together with pathological examination, serves as the key diagnostic tool in IBD, is further found useful in the monitoring of the disease activity. As mentioned before, endoscopy for MH evaluation is an invasive, time-consuming, and expensive technique. Non-invasive diagnostic biomarkers, able to at least limit the number of endoscopies, are searched for intensively. The urgent requirement for non-invasive indices in IBD is additionally augmented by the recent global growth in IBD incidence rates [24]. Furthermore, the current COVID-19 pandemic highlighted the significance of non-invasive point-of-care tests, that may be integrated with telemedicine in IBD patient care [25,26,27].

From among numerous potential biomarkers which have been evaluated in IBD, only faecal calprotectin (FC) has the potential to serve as an indicator of IBD activity [28].

Calprotectin is a cytosolic protein which is present in high concentrations in human neutrophils. Lower concentrations are found in monocytes and reactive macrophages. It has antimicrobial activity, as it sequesters zinc ions, helping to outcompete bacteria and yeast for this element and thus limiting their growth. The release of calprotectin is most likely related with death of neutrophils at the site of inflammation [29]. In the context of IBD, calprotectin release in the intestine translates into elevated concentrations in stool. As such, FC can be regarded as a measure of neutrophil infiltration of the intestinal mucosa and a marker of the overall severity of gut inflammation.

Faecal calprotectin is stable for 4–7 days [30] and the sampling does not involve uncomfortable procedures. In addition to specialised diagnostic laboratory protocols for FC determination, there are tests designed for low throughput point-of-care analysis, as well as self-testingthat can be performed at home by a patient. The latter assay kits are supported by a mobile device application, which helps to read out, calculate and communicate the result to a physician. This could be a useful tool for disease monitoring, prediction of relapses and for therapy optimisation. 

Therefore, we conducted this meta-analysis to answer the questions: what is the diagnostic accuracy of the FC test as a biomarker of mucosal healing in IBD, and could it be applied in disease monitoring as well as in the assessment of the effectiveness of an ongoing therapy.

## 2. Materials and Methods

The search for studies on the use of faecal calprotectin as a biomarker of mucosal healing in ulcerative colitis and Crohn’s disease utilised the following strategy: (faecal calprotectin) AND (mucosal healing). In each round, the latter term was replaced with one of the following: healing, endoscopy, colonoscopy, Crohn’s disease, ulcerative colitis, IBD, or inflammatory bowel disease. Spelling variants were included. The publication dates were constrained to studies published between 1 January 2009 and 31 August 2020. The searched databases were PubMed and Scopus. 

Query results were cross-searched and cleaned of duplicates. The selection process was composed of the following steps: title search, abstract screening, full text search, and eligible study data extraction. At each step, study selection was verified by a second investigator. The inclusion criteria were: original publication, publication in English language, mucosal healing was diagnosed according to specific standards (indices); studies provided sufficient data to reconstruct contingency matrices. Exclusion criteria were as follows: letters, editorials, comments, conference papers; paediatric or adolescent patients; non-IBD related; non-colonic Crohn’s disease; studies performed on animals or tissue cultures; experimental studies. 

We have extracted information from eligible studies based on the first author, year of publication, research object, definition of mucosal healing, population size, prevalence, cut-off values, FC measurement method, and true and false positives and negatives. In two cases authors were asked to rectify their data before being included in the data set (Table S1).

The analysis of data was performed with R (version 4.0.2, downloaded: 22 June 2020). The packages, meta (univariate random effects meta-analysis, funnel plots) and mada (sROC, bivariate effects analysis with Reitsma model) were used. The summary statistics (sensitivity, specificity, and DOR) were analysed by performing univariate analysis with the meta package. The “metabin” function was used to calculate the DOR from contingency tables with the inverse variance method for weights of individual studies. Forrest and funnel plots were generated with the meta package. The mada package for bivariate analysis was applied in the estimation of the sROC curve. For this purpose, the “reitsma” function was used; with correction for zero values in rows only. The function estimated variance components by the restricted maximum likelihood method. We calculated the pooled sensitivity, specificity, and diagnostic odds ratio with their 95% CI. Statistical heterogeneity was assessed with Cochrane’s Q statistic and Higgins’ *I*^2^ value.

## 3. Results

This study was performed and reported according to the statement on Preferred Reporting Items for Systematic Reviews and Meta-Analyses (PRISMA) [31]. More details are presented in the supplementary PRISMA Checklist (Table S2). The two literature databases contained 2305 unique records which fit the search strategy. This formed a title search pool from which 562 publications were selected for the abstract screening. Out of these, 106 were found eligible after the screening of abstracts. Of that number, 52 publications contained information required for the meta-analysis (Figure S1: data acquisition flow diagram). Eventually, data from 16 publications were included in the data set on the use of FC in Crohn’s disease. The diagnostic test for FC in ulcerative colitis was described in 35 original publications. In several cases, more than one data set was extracted from a publication. This was the case for studies in which different mucosal healing definitions or different FC measurement kits were compared. 

**Figure 1:**
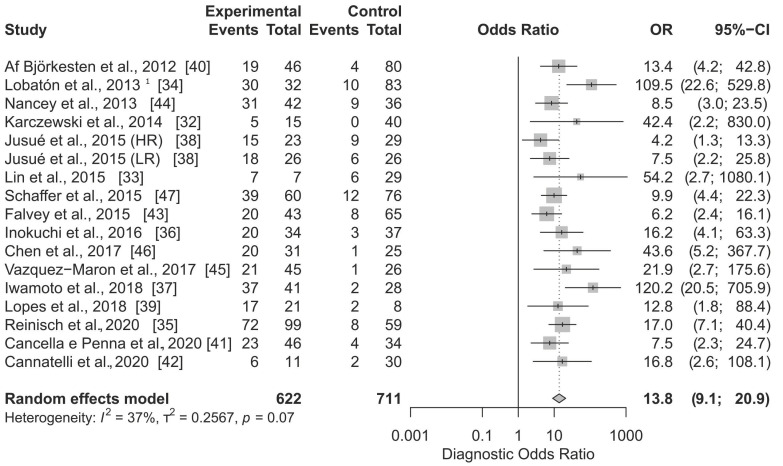
Forest plot of Diagnostic Odds Ratio calculated for the use of FC as the biomarker of mucosal healing in Crohn’s disease. Abbreviations: CI, confidence interval; OR, odds ratio. Notes: Jusué et al. compared High Range (HR) and Low Range (LR) Quantum Blue rapid kits; ^1^ ELISA test.

In Figure 1 the total effect sizes of all 17 applications are shown. The diagnostic OR of the random effect model is 13.8 (95% CI, 9.1 to 20.9) and the *p*-value < 0.0001. With the use of FC in the diagnosis of MH in Crohn’s disease, the odds of apositive result among persons with the disease in remission is approximately 14 times higher than the odds for positive results among persons with still active disease. The Higgins’ *I*^2^ of all studies is 37%, and the *p*-value of the Cochrane Q statistic is 0.07, indicating that there is moderate heterogeneity.

Table 1 shows the calculated sensitivity of the FC diagnostics of mucosal healing, with grouping according to the applied definition. The summary sensitivity with the random effect model is 0.828 (95% CI, 0.769 to 0.874). The Higgins’ *I*^2^ = 51.7% and the Cochrane Q statistic is 36.18 (*p*-value = 0.0027) which suggest the existence of moderatetosubstantial heterogeneity. There is no significant difference in sensitivity between the SES-CD-based definitions of mucosal healing. The highest cumulative sensitivity was calculated for SES-CD ≤ 2, that is 0.841 (5 studies). The summary specificity of 17 compared applications was estimated to be 0.759 (95% CI, 0.683 to 0.821). The Higgins’ *I*^2^ = 80.2% and the Cochrane Q statistic is 75.41 (*p*-value < 0.0001) which is indicative of substantial heterogeneity between the studies.

**Figure 2:**
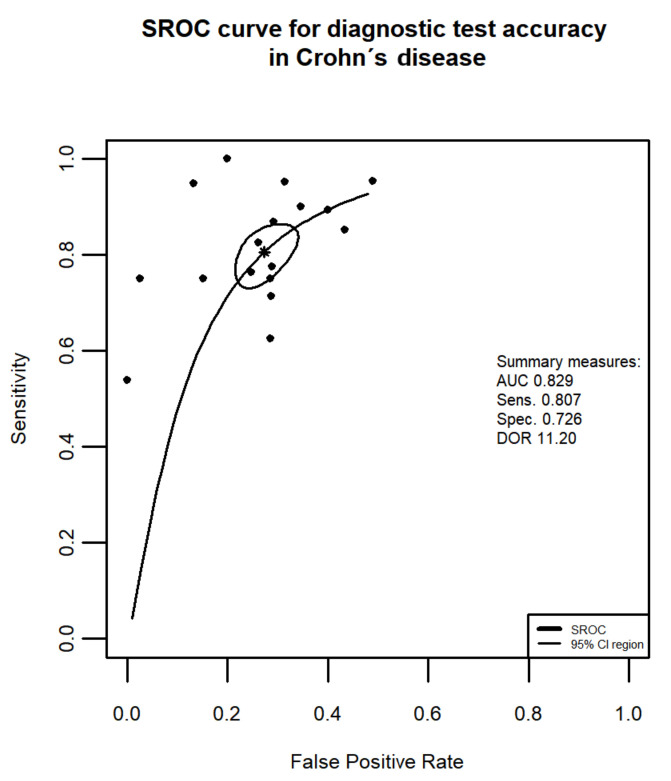
Summary Receiver Operating Characteristic (SROC) curve of the application of FC in the diagnosis of mucosal healing in Crohn’s disease superimposed on Sensitivity and False Positive Rate (1-Specificity) results from 17 studies. Summary Sensitivity and summary False Positive Rate are shown as a star, with the 95% CI area circled. Abbreviations: AUC, area under the curve; Sens., sensitivity; Spec., specificity; DOR, diagnostic odds ratio, CI, confidence interval.

Figure 2 presents the sROC curve for the application of FC in the diagnosis of MH in Crohn’s disease. The summary’s AUC is 0.829 and the bivariate model-based estimation of DOR = 11.20. 

To verify if there might be a publication bias, the DOR was plotted against the standard error of the effect estimate (data not shown). In the obtained funnel plot, three out of 17 included in this meta-analysis of applications of FC to diagnose MH, are found outside of the 95% (*p*-value 0.05) boundaries. These were Jusué et al. [38] with a High Range test kit, Lobatón et al. [34], and Iwamoto et al. [37]. A tendency for a higher DOR in studies with a smaller tested population was observed.

**Figure 3:**
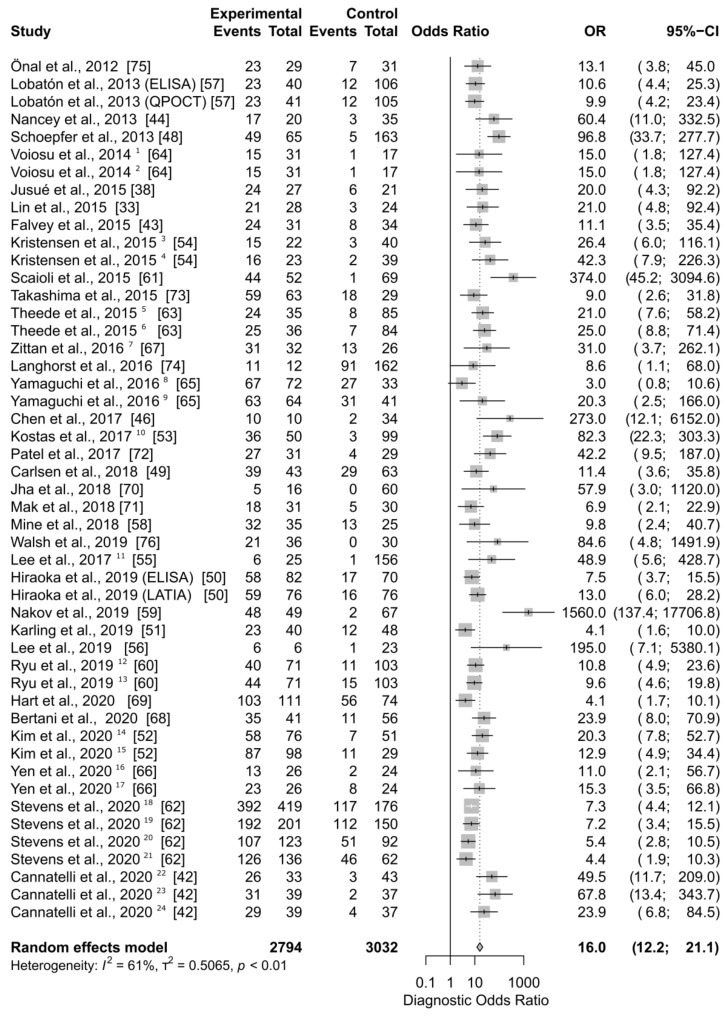
Forest plot of Diagnostic Odds Ratio calculated for the use of FC as the biomarker of mucosal healing in ulcerative colitis. Abbreviations: CI, confidence interval; OR, odds ratio. Notes: Hiraoka et al. compared ELISA and latex agglutination turbidimetric immunoassay (LATIA) kits; Lobatón et al. compared ELISA and Q-Point Of Care Test (QPOCT); Further indexed numbers mark different MH definitions: ^1^ MES = 0; ^2^ SES-CD ≤ 3; ^3^ MES = 0; ^4^ MES ≤ 1; ^5^ MES = 0; ^6^ UCEIS = 0; ^7^ MES = 0; ^8^ MES = 0; ^9^ MES ≤ 1; ^10^ MES = 0; ^11^ MES = 0; ^12^ MES = 0; ^13^ UCEIS ≤ 1.; ^14^ MES = 0; ^15^ MES ≤ 1; ^16^ MES = 0; ^17^ MES ≤ 1; ^18^ MES ≤ 1 (8 weeks of therapy); ^19^ MES ≤ 1 (52 weeks of therapy); ^20^ MES = 0 (8 weeks of therapy); ^21^ MES = 0 (52 weeks of therapy); ^22^ MES = 0, ^23^ UCEIS ≤ 1; ^24^ modified PICaSSO ≤ 3.

Figure 3 shows DOR sizes of 49 applications of FC in the diagnosis of mucosal healing in ulcerative colitis. The summary diagnostic OR of the univariate random effect model is 16.0 (95% CI, 12.2 to 21.1) and the *p*-value < 0.0001. The calculated cumulative OR for the use of FC in the diagnosis of MH in UC means that the odds of a positive result among persons with the disease in remission is 16 times higher than the odds for positive results among persons with still active inflammation. The Higgins’ *I*^2^ of all studies is 61%, and the *p*-value of the Cochrane Q statistic is <0.0001, indicating that there is moderatetosubstantial heterogeneity in the compared studies.

In Table 2, the FC diagnostic test sensitivity and specificity values in UC calculated from contingency matrix data are presented. The summary sensitivity with the univariate random effect model is 0.804 (95% CI, 0.757 to 0.843). The Higgins’ I^2^ = 87.5% and the Cochrane Q statistic is 363.28 (*p*-value < 0.0001) which suggest the existence of substantial heterogeneity. The MES = 0 as the definition of MH was applied in 24 studies for which the specificity was estimated to be 0.798 (95% CI 0.743; 0.843). Eleven studies applied MES ≤ 1 and for those studies the test’s specificity was 0.766 (95% CI 0.697; 0.823). The summary specificity of 49 compared applications of the FC was estimated to be 0.817 (95% CI, 0.780 to 0.848). The Higgins’ I^2^ = 78.6% and the Cochrane Q statistic is 209.42 (*p*-value < 0.0001), indicating substantial heterogeneity between the studies of FC in the detection of the mucosal healing.

**Figure 4:**
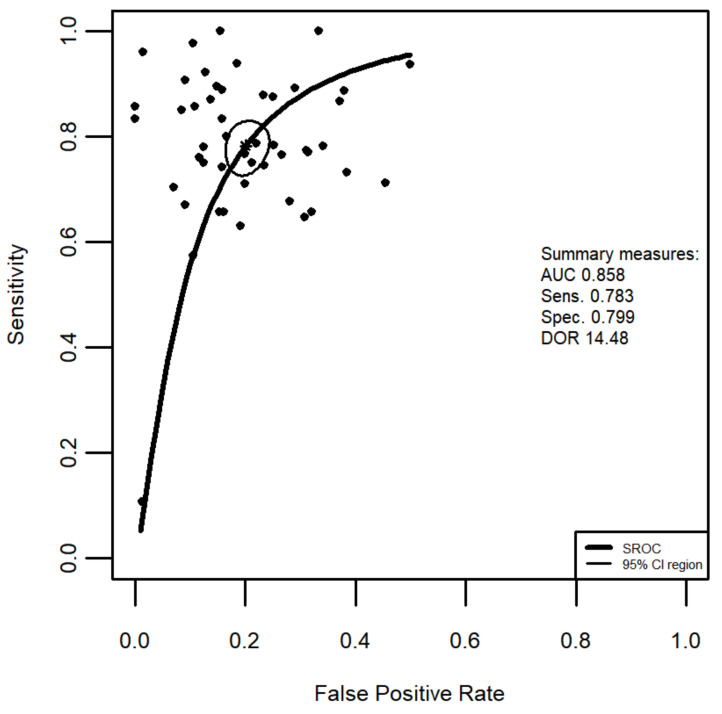
Summary Receiver Operating Characteristic (sROC) curve of the application of FC in the diagnosis of mucosal healing in ulcerative colitis superimposed on Sensitivity and False Positive Rate (1-specificity) results from 49 applications. Summary Sensitivity and summary False Positive Rates are shown as a star, with the 95% CI area circled. Abbreviations: AUC, area under the curve; Sens., sensitivity; Spec. specificity; DOR, diagnostic odds ratio, CI, confidence interval.

Figure 4 shows a summary ROC curve (plot of sensitivity against 1-specificity) which was estimated with the use of the bivariate random effects meta-analysis model. The summary ROC curve was generated with 49 studies/applications of FC in the diagnosis of mucosal healing in ulcerative colitis. The summary test sensitivity is 0.783 (95% CI 0.738 to 0.822), while the specificity of the use of faecal calprotectin was estimated to be 0.799 (95% CI 0.769 to 0.829). The summary’s AUC is 0.858 and the bivariate model-based estimation of DOR = 14.48. This is less than the estimation based on the univariate random effects model. 

To study possible bias in reporting, a funnel plot of the diagnostic OD against the standard error of the effect estimate was generated (data not shown). Eleven studies out of 49 included in this meta-analysis are found outside the 95% (*p*-value 0.05) boundaries of the funnel plot. The distribution was skewed towards the studies with a relatively small sample size and high DOR. Four of those are still within the boundaries. Most of the studies lie in the high significance region, suggesting that the asymmetry is not due the publication selection. Studies outside the funnel area: Schoepfers et al. [48], Scaioli et al. [61], Yamaguchi et al. [65], Kostas et al. [53], Nakov et al. [59], Karling et al. [51], Hart et al. [69], and all application studies contained in the publication of Stevens et al. [62]. 

## 4. Discussion

The diagnostic odds ratio is the main measure for comparison of the diagnostic tests in this meta-analysis. Different though similar results could be observed with the use of two DOR calculation models. The univariate effects model predicts the DOR to be higher than that estimated with the use of the bivariate model based on the Reitsma’s method [77]. This is true for the application of FC as the biomarker of MH in both CD and UC. A DOR estimated with either method can be found within a confidence interval of the other. Both DOR values generally represent good diagnostic accuracy of the test. Thus, results of our meta-analysis support the use of faecal calprotectin as a non-invasive biomarker of mucosal healing in IBD.

The calculated summary DOR of faecal calprotectin-based determination of a patient’s mucosal healing status in CD, shows that the odds of a positive result (low faecal calprotectin) among patients with the disease in remission is 13.8 (95% CI 9.1–20.9) times higher than the odds for positive results among patients with still active inflammation (Figure 1). The Reitsma’s model estimates the DOR to be 11.48 (Figure 3). The DOR in the case of FC applied in UC is higher: 16.0 and 14.48 (for univariate and bivariate models, respectively). Only in one study, by Yamaguchi et al. [65], did the estimated DOR’s lower boundary of the 95% confidence interval drop below 1 (Figure 3). This was observed when MES = 0 was used as the definition of MH. On the other hand, the summary DOR could be artificially elevated/biased due to a high DOR (DOR = 1560), as obtained from the study of Nakov et al. [59]. It is worth noting four applications of FC as a marker of mucosal healing were presented in the publication by Stevens et al. [62]. Authors collected data from hundreds of UC patients participating in a phase 4 trial and performed a post-hoc analysis with two MH definitions (MES = 0, MES ≤ 1). These were applied to two datasets; endoscopic evaluation of mesalamine treatment at week 8, and endoscopic evaluation of mesalamine maintenance treatment effects at week 52. Based on these data from Stevens et al. [62], all four individual test DOR values were rather low (between 4.4 and 7.3). In fact, none of the DOR confidence intervals even reached the cumulative value (DOR = 16.0) estimated in this study. This can be interpreted as follows: DOR estimated for tests with larger populations might result in lower values of the summary DOR than estimated here. This notion is supported by our bias analysis; studies with high SE tend to have a higher estimated OR. 

The overall good diagnostic test accuracy can be seen from the summary ROC of the FC diagnostic test. In patients with CD the area under the summary ROC covers 82.9% of the plot area, whereas in patients with UC the area under the sROC = 85.8%. 

One of the issues which limits this meta-analysis is the lack of agreement on the definition of mucosal healing in IBD patients. In our analysis there was no difference of the DOR between studies classified by their MH definition (both for UC and CD; data not shown). The MH in ulcerative colitis in the compared studies was determined with the use of 6scales/indices which took 13 different values. The most commonly used was the Mayo Endoscopic Score. It was applied in 37 studies with the majority (24 studies) defining MH as MES = 0. In one study it was defined as MES ≤ 2 [46]. Other MH definitions used were the modified Baron Score (mBS = 0, mBS ≤ 1), the modified PICaSSO ≤ 3 [42], the Rachmilewitz Index (RI ≤ 1 [74], RI ≤ 2 [44], RI ≤ 4 [75]); the Simple Endoscopic Score for Ulcerative Colitis (SES-UC ≤ 3 [64]), and the Ulcerative Colitis Endoscopic Index of Severity (UCEIS = 0 [63], UCEIS ≤ 1 [42,60,76], UCEIS ≤ 3 [33]).

In the case of CD in included studies, MH was defined as SES-CD = 0, SES-CD ≤ 2, SES-CD ≤ 3, as well as CDEIS < 3, CDEIS ≤ 3, and CDEIS < 6 (Table 1 and references therein). SES-CD was the most often used index (total 13 studies). This could be explained by the fact that SES-CD is a simplified, easier to calculate version of CDEIS. Travis et al. performed a validation test in which the SES-CD and CDEIS were compared. Both scores showed a strong positive correlation (r = 0.92) [78]. 

The studies included in this meta-analysis show the wide range of cut-off values (13.9 to 251 μg/g) which were chosen by authors studying FC as a marker of mucosal healing in ulcerative colitis. The most common thresholds are found between 150–250 μg/g (31/49 studies). Similarly, there was no universal cut-off value for FC in analysed studies on CD. The reported range is very wide: 54–918 μg/g FC, mean cut-off 205 μg/g. The cut-off between 150 μg/g and 250 μg/g was used in 7 out of 17 studies. The authors of studies with the extreme cut-off values used different definitions of endoscopic mucosal healing (SES-CD = 0 and CDEIS <6, respectively), as well as applied different types of tests for FC in stool samples (rapid test and ELISA, respectively) [33,38]. According to Moniuszko et al.,rapid FC tests which could be performed at the point-of-care yielded results in high agreement with ELISA-assays [79].

IBD cannot be seen as the exclusive cause of elevated faecal calprotectin. In a study by Meuccion et al., 36% of patients with normal colonoscopy had elevated FC levels, and the marker was elevated in a similar proportion of those with trivial endoscopic findings [80]. Moreover, faecal calprotectin above 50 μg/g has been found in 85% of patients with colonic cancer, 81% of those with “inflammatory conditions” (active CD or in remission, active UC, ischaemic colon), and 50% of those with UC in remission [80]. Furthermore, common medications are associated with increased FC. Lundgren et al. observed that among patients with a normal colonoscopy, FC above 50 μg/g was shown for 55% patients using acetylsalicylic acid, 24% using non-steroidal anti-inflammatory drugs, and 52% treated with proton pump inhibitors [81]. On the other hand, a recent multicenter study on pregnant women concluded that physiological changes due to pregnancy do not affect FC levels [82]. Therefore, Julsgaard et al. suggested the use of FC in the monitoring of IBD during pregnancy [82]. The lack of a universal FC cut-off is one of the biggest limitations of the use of this biomarker in either UC or CD. The issue has several factors influencing it: the methodological and technical differences between FC assays, the MH definition applied by physicians, and inter-individual variability in FC values. As for the latter, a potential explanation for the wide ranges in reporting cut-off values might be residual inflammation that may still remain at a microscopic (histologic; neutrophiles infiltrating the mucosa) level despite the remission and mucosal healing being reported by an endoscopist.

The following limitations of this meta-analysis should be taken in consideration. Firstly, due to apparent fluctuations of cut-off values for FC in analysed studies, this meta-analysis could not provide a clinical recommendation on one definite FC cut-off value which could help in the assessment of IBD activity without an endoscopy. Secondly, obvious heterogeneity existed across the included studies. The different MH definitions, methodological differences between FC assays, as well as sizes of tested populations might be the sources of heterogeneity in the meta-analysis.

## 5. Conclusions

In the light of the obtained results, a positive answer to the main question of the meta-analysis can be given. The DOR for the use of FC in CD is estimated to be 11.20. In the case of UC, the DOR is 14.48. The bivariate model applied to the estimation of sensitivity of FC in the detection of mucosal healing resulted in the summary sensitivity of 0.807 and 0.783 for CD and UC, respectively. Despite good sensitivity and specificity of the FC test in the determination of IBD activity, we suggest caution in clinical decision-making on a single FC result. Nevertheless, FC is the most widely used and the most supported tool in the assessment of mucosal healing in UC and CD without the need for endoscopy. The odds of confirming mucosal healing with a non-invasive FC test support their further use in the management of IBD. We recommend that future studies reporting on the topic should include data (e.g., contingency tables) for various cut-off values. Further research into the establishment of a universal cut-off for FC should accompany work into the interchangeability of FC diagnostic tests.

## Figures and Tables

**Table 1:** Diagnostic test sensitivity and specificity calculated for the use of FC as the biomarker of mucosal healing in Crohn’s disease. Studies are grouped by the MH definition applied. Abbreviations: CDEIS, Crohn’s Disease Endoscopic Index of Severity; CI, confidence interval; MH, mucosal healing; SES-CD, Simple Endoscopic Score for Crohn’s Disease; Sens., sensitivity; Spec., specificity. Notes: Jusué et al. compared High Range (HR) and Low Range (LR) Quantum Blue rapid kits; ^1^ ELISA test.

Author, Year of Publication	Ref.	Sens. (95%-CI)	Spec. (95%-CI)	Mucosal Healing Definition
Karczewski et al., 2014	[32]	1.000 (0.478; 1.000)	0.800 (0.663; 0.900)	CDEIS < 3
Lin et al., 2015	[33]	0.538 (0.251; 0.808)	1.000 (0.852; 1.000)	CDEIS < 6
Lobatón et al., 2013 ^1^	[34]	0.750 (0.588; 0.873)	0.973 (0.907; 0.997)	CDEIS ≤ 3
Reinischet al., 2020	[35]	0.900 (0.812; 0.956)	0.654 (0.538; 0.758)	CDEIS ≤ 3
Inokuchi et al., 2016	[36]	0.870 (0.664; 0.972)	0.708 (0.559; 0.830)	SES-CD = 0
Iwamoto et al., 2018	[37]	0.949 (0.827; 0.994)	0.867 (0.693; 0.962)	SES-CD = 0
Jusué et al., 2015 (HR)	[38]	0.625 (0.406; 0.812)	0.714 (0.513; 0.868)	SES-CD = 0
Jusué et al., 2015 (LR)	[38]	0.750 (0.533; 0.902)	0.714 (0.513; 0.868)	SES-CD = 0
Lopes et al., 2018	[39]	0.895 (0.669; 0.987)	0.600 (0.262; 0.878)	SES-CD = 0
Af Björkesten et al., 2012	[40]	0.826 (0.612; 0.950)	0.738 (0.642; 0.820)	SES-CD ≤ 2
Cancella e Penna et al., 2020	[41]	0.852 (0.663; 0.958)	0.566 (0.423; 0.702)	SES-CD ≤ 2
Cannatelli et al., 2020	[42]	0.750 (0.349; 0.968)	0.848 (0.681; 0.949)	SES-CD ≤ 2
Falvey et al., 2015	[43]	0.714 (0.513; 0.868)	0.713 (0.600; 0.808)	SES-CD ≤ 2
Nancey et al., 2013	[44]	0.775 (0.615; 0.892)	0.711 (0.541; 0.846)	SES-CD ≤ 2
Vazquez-Morón et al., 2017	[45]	0.955 (0.772; 0.999)	0.510 (0.363; 0.656)	SES-CD ≤ 2
Chen et al., 2017	[46]	0.952 (0.762; 0.999)	0.686 (0.507; 0.831)	SES-CD ≤ 3
Schaffer et al., 2015	[47]	0.765 (0.625; 0.872)	0.753 (0.647; 0.840)	SES-CD ≤ 3
Random effects model:		0.828 (0.769; 0.874)	0.759 (0.683; 0.821)	
Quantifying heterogeneity:				
		tau^2^ = 0.2803	tau^2^ = 0.4648	
		*I*^2^ = 51.7%	*I*^2^ = 80.2%	
		Q = 36.18 (0.0027)	Q = 75.41 (<0.0001)	

**Table 2:** Diagnostic test sensitivity and specificity calculated for the use of FC as the biomarker of mucosal healing in ulcerative colitis. Studies are grouped by the MH definition applied. Abbreviations: CI, confidence interval; mBS, modified Baron Score; MES, Mayo endoscopic subscore; MH, mucosal healing; RI, Rachmilewitz Index; SES-CD, Simple Endoscopic Score for Ulcerative Colitis;Sens, sensitivity; Spec, specificity;UCEIS, Ulcerative Colitis Endoscopic Index of Severity. Notes: Stevens et al. compared diagnostic tests after 8 and 52 weeks of treatment (wk 8 andwk52, respectively). Hiraoka et al. compared ELISA and latex agglutination turbidimetric immunoassay (LATIA) kits; Lobatón et al. compared ELISA and Q-Point Of Care Tests (QPOCT).

Author, Year of Publication	Ref.	Sens. (95%-CI)	Spec. (95%-CI)	Mucosal Healing Definition
Falvey et al., 2015	[43]	0.750 (0.566; 0.885)	0.788 (0.611; 0.910)	mBS = 0
Schoepfer et al., 2013	[48]	0.907 (0.797; 0.969)	0.908 (0.855; 0.947)	mBS ≤ 1
Cannatelli et al., 2020	[42]	0.897 (0.726; 0.978)	0.851 (0.717; 0.938)	MES = 0
Carlsen et al., 2018	[49]	0.574 (0.448; 0.693)	0.895 (0.752; 0.971)	MES = 0
Hiraoka et al., 2019 (ELISA)	[50]	0.773 (0.662; 0.862)	0.688 (0.573; 0.789)	MES = 0
Hiraoka et al., 2019 (LATIA)	[50]	0.787 (0.677; 0.873)	0.779 (0.670; 0.866)	MES = 0
Jusué et al., 2015	[38]	0.800 (0.614; 0.923)	0.833 (0.586; 0.964)	MES = 0
Karling et al., 2019	[51]	0.657 (0.478; 0.809)	0.679 (0.537; 0.801)	MES = 0
Kim et al., 2020	[52]	0.892 (0.791; 0.956)	0.710 (0.581; 0.818)	MES = 0
Kostas et al., 2017	[53]	0.923 (0.791; 0.984)	0.873 (0.796; 0.929)	MES = 0
Kristensen et al., 2015	[54]	0.833 (0.586; 0.964)	0.841 (0.699; 0.934)	MES = 0
Lee et al., 2017	[55]	0.857 (0.421; 0.996)	1.000 (0.846; 1.000)	MES = 0
Lee et al., 2019	[56]	0.857 (0.421; 0.996)	0.891 (0.835; 0.933)	MES = 0
Lobatón et al., 2013 (ELISA)	[57]	0.657 (0.478; 0.809)	0.847 (0.766; 0.908)	MES = 0
Lobatón et al., 2013 (QPOCT)	[57]	0.657 (0.478; 0.809)	0.838 (0.756; 0.901)	MES = 0
Mine et al., 2018	[58]	0.711 (0.557; 0.836)	0.800 (0.519; 0.957)	MES = 0
Nakov et al., 2019	[59]	0.960 (0.863; 0.995)	0.985 (0.918; 1.000)	MES = 0
Ryu et al., 2019	[60]	0.784 (0.647; 0.887)	0.748 (0.662; 0.822)	MES = 0
Scaioli et al., 2015	[61]	0.978 (0.882; 0.999)	0.895 (0.803; 0.953)	MES = 0
Stevens et al., 2020 (wk 8)	[62]	0.677 (0.598; 0.749)	0.719 (0.585; 0.830)	MES = 0
Stevens et al., 2020 (wk 52)	[62]	0.733 (0.660; 0.797)	0.615 (0.406; 0.798)	MES = 0
Theede et al., 2015	[63]	0.750 (0.566; 0.885)	0.875 (0.787; 0.936)	MES = 0
Voiosu et al., 2014	[64]	0.938 (0.698; 0.998)	0.500 (0.319; 0.681)	MES = 0
Yamaguchi et al., 2016	[65]	0.713 (0.610; 0.801)	0.545 (0.234; 0.833)	MES = 0
Yen et al., 2020	[66]	0.867 (0.595; 0.983)	0.629 (0.449; 0.785)	MES = 0
Zittan et al., 2016	[67]	0.705 (0.548; 0.832)	0.929 (0.661; 0.998)	MES = 0
Bertani et al., 2020	[68]	0.761 (0.612; 0.874)	0.882 (0.761; 0.956)	MES ≤ 1
Hart et al., 2020	[69]	0.648 (0.568; 0.722)	0.692 (0.482; 0.857)	MES ≤ 1
Jha et al., 2018	[70]	1.000 (0.478; 1.000)	0.845 (0.740; 0.920)	MES ≤ 1
Kim et al., 2020	[52]	0.888 (0.808; 0.943)	0.621 (0.423; 0.793)	MES ≤ 1
Kristensen et al., 2015	[54]	0.889 (0.653; 0.986)	0.841 (0.699; 0.934)	MES ≤ 1
Mak et al., 2018	[71]	0.783 (0.563; 0.925)	0.658 (0.486; 0.804)	MES ≤ 1
Patel et al., 2017	[72]	0.871 (0.702; 0.964)	0.862 (0.683; 0.961)	MES ≤ 1
Stevens et al., 2020 (wk 8)	[62]	0.770 (0.731; 0.806)	0.686 (0.577; 0.782)	MES ≤ 1
Stevens et al., 2020 (wk 52)	[62]	0.632 (0.575; 0.686)	0.809 (0.667; 0.909)	MES ≤ 1
Takashima et al., 2015	[73]	0.766 (0.656; 0.855)	0.733 (0.449; 0.922)	MES ≤ 1
Yamaguchi et al., 2016	[65]	0.670 (0.566; 0.764)	0.909 (0.587; 0.998)	MES ≤ 1
Yen et al., 2020	[66]	0.742 (0.554; 0.881)	0.842 (0.604; 0.966)	MES ≤ 1
Chen et al., 2017	[46]	0.833 (0.516; 0.979)	1.000 (0.891; 1.000)	MES ≤ 2
Cannatelli et al., 2020	[42]	0.879 (0.718; 0.966)	0.767 (0.614; 0.882)	modPICaSSO ≤ 3
Langhorst et al., 2016	[74]	0.108 (0.055; 0.185)	0.986 (0.925; 1.000)	RI ≤ 1
Nancey et al., 2013	[44]	0.850 (0.621; 0.968)	0.914 (0.769; 0.982)	RI ≤ 2
Önal et al., 2012	[75]	0.767 (0.577; 0.901)	0.800 (0.614; 0.923)	RI ≤ 4
Voiosu et al., 2014	[64]	0.938 (0.698; 0.998)	0.500 (0.319; 0.681)	SES-UC ≤ 3
Theede et al., 2015	[63]	0.781 (0.600; 0.907)	0.875 (0.787; 0.936)	UCEIS = 0
Cannatelli et al., 2020	[42]	0.939 (0.798; 0.993)	0.814 (0.666; 0.916)	UCEIS ≤ 1
Ryu et al., 2019	[60]	0.746 (0.616; 0.850)	0.765 (0.677; 0.839)	UCEIS ≤ 1
Walsh et al., 2019	[76]	1.000 (0.839; 1.000)	0.667 (0.510; 0.800)	UCEIS ≤ 1
Lin et al., 2015	[33]	0.875 (0.676; 0.973)	0.750 (0.551; 0.893)	UCEIS ≤ 3
Random effects model:		0.804 (0.757; 0.843)	0.817 (0.780; 0.848)	
Quantifying heterogeneity:				
		tau^2^ = 0.6772	tau^2^ = 0.4718	
		*I*^2^ = 87.5%	*I*^2^ = 78.6%	
		Q = 363.28 (<0.0001)	Q = 209.42 (<0.0001)	

## Data Availability

Data used in this meta-analysis are contained in Supplementary Materials.

## Supplementary Materials

The following are available online at https://www.mdpi.com/article/10.3390/jcm10102203/s1, Figure S1: Flow diagram of data selection; Table S1: Meta-analysis data; Table S2: PRISMA 2009 Checklist.
