# Supplementary material for: Faecal Calprotectin in Assessment of Mucosal Healing in Adults with Inflammatory Bowel Disease: A Meta-Analysis
Source: J Clin Med. 2021 May 19;10(10):2203. doi: 10.3390/jcm10102203 (PMC8161009; doi:10.3390/jcm10102203)

Table S1. Meta-analysis data set. Abbreviations: MH, mucosal healing; sens, sensitivity; spec, specificity; TP, true positives; TN, true negatives; FP, false positives, FN, false negatives.

CROHN'S DISEASE DATA SET

**Study**	**MH_definition**	**MH_cases**	**N**	**cutoff**	**sens**	**spec**	**TP**	**FN**	**TN**	**FP**
Af Björkesten et al., 2012	SES-CD≤2	23	126	100	0.84	0.74	19	4	76	27
Lobatón et al., 2013	CDEIS≤3	40	115	274	0.76	0.97	30	10	73	2
Nancey et al., 2013	SES-CD≤2	40	78	250	0.78	0.71	31	9	27	11
Karczewski et al., 2014	CDEIS<3	5	55	76	0.96	0.8	5	0	40	10
Jusué et al., 2015 (HR)	SES-CD=0	24	52	54	0.63	0.71	15	9	20	8
Jusué et al., 2015 (LR)	SES-CD=0	24	52	122	0.75	0.71	18	6	20	8
Lin et al., 2015	CDEIS<6	13	36	918	0.5	0.99	7	6	23	0
Schaffer et al., 2015	SES-CD≤3	51	136	250	0.76	0.75	39	12	64	21
Falvey et al., 2015	SES-CD≤2	28	108	125	0.71	0.71	20	8	57	23
Inokuchi et al., 2016	SES-CD=0	23	71	180	0.87	0.71	20	3	34	14
Chen et al., 2017	SES-CD≤3	21	56	250	0.93	0.7	20	1	24	11
Vazquez-Maron et al., 2017	SES-CD≤2	22	71	71	0.96	0.52	21	1	25	24
Iwamoto et al., 2018	SES-CD=0	39	69	92	0.94	0.88	37	2	26	4
Lopes et al., 2018	SES-CD=0	19	29	100	0.92	0.65	17	2	6	4
Reinisch et al. 2020	CDEIS≤3	80	156	250	0.9	0.65	72	8	51	27
Cancella e Penna et al. 2020	SES-CD≤2	27	80	273	0.87	0.56	23	4	30	23
Cannatelli et al. 2020	SES-CD≤2	8	41	96	0.75	0.844	6	2	28	5

ULCERATIVE COLITIS DATA SET

**Study**	**MH_definition**	**MH_cases**	**N**	**cutoff**	**sens**	**spec**	**TP**	**FN**	**TN**	**FP**
Önal et al., 2012	RI≤4	30	60	99.5	0.77	0.79	23	7	24	6
Lobatón et al., 2013 (ELISA)	MES=0	35	146	160	0.67	0.85	23	12	94	17
Lobatón et al., 2013 (QPOCT)	MES=0	35	146	160	0.65	0.84	23	12	93	18
Nancey et al., 2013	RI≤2	20	55	250	0.87	0.91	17	3	32	3
Schoepfers et al., 2013	mBS≤1	54	228	57	0.9	0.91	49	5	158	16
Voiosu et al., 2014	MES=0	16	48	30	0.94	0.5	15	1	16	16
Voiosu et al., 2014	SES≤3	16	48	30	0.94	0.5	15	1	16	16
Jusué et al., 2015	MES=0	30	48	50	0.79	0.85	24	6	15	3
Lin et al., 2015	UCEIS≤3	24	52	918	0.88	0.75	21	3	21	7
Falvey et al., 2015	mBS=0	32	65	125	0.74	0.8	24	8	26	7
Kristensen et al., 2015	MES=0	18	62	61	0.84	0.83	15	3	37	7
Kristensen et al., 2015	MES≤1	18	62	96	0.91	0.83	16	2	37	7
Scaioli et al., 2015	MES=0	45	121	110	0.98	0.9	44	1	68	8
Takashima et al., 2015	MES≤1	77	92	200	0.77	0.72	59	18	11	4
Theede et al., 2015	MES=0	32	120	192	0.75	0.88	24	8	77	11
Theede et al., 2015	UCEIS=0	32	120	192	0.79	0.87	25	7	77	11
Zittan et al., 2016	MES=0	44	58	100	0.71	0.91	31	13	13	1
Langhorst et al., 2016	RI≤1	102	174	13.9	0.11	0.99	11	91	71	1
Yamaguchi et al., 2016	MES=0	94	105	194	0.71	0.53	67	27	6	5
Yamaguchi et al., 2016	MES≤1	94	105	200	0.67	0.91	63	31	10	1
Chen et al., 2017	MES≤2	12	44	250	0.85	0.999	10	2	32	0
Kostas et al., 2017	MES=0	39	149	174	0.92	0.87	36	3	96	14
Patel et al., 2017	MES≤1	31	60	60	0.86	0.87	27	4	25	4

Carlsen et al., 2018	MES=0	68	106	25	0.58	0.9	39	29	34	4
Jha et al., 2018	MES≤1	5	76	158	0.9	0.85	5	0	60	11
Mak et al., 2018	MES≤1	23	61	250	0.77	0.67	18	5	25	13
Mine et al., 2018	MES=0	45	60	201	0.71	0.78	32	13	12	3
Walsh et al., 2019	UCEIS≤1	21	66	187	0.999	0.67	21	0	30	15
Lee et al., 2019 *2	MES=0	7	181	187	0.86	0.89	6	1	155	19
Hiraoka et al., 2019 (ELISA)	MES=0	75	152	184	0.78	0.69	58	17	53	24
Hiraoka et al., 2019 (LATIA)	MES=0	75	152	224	0.79	0.78	59	16	60	17
Nakov et al., 2019	MES=0	50	116	99	0.97	0.98	48	2	65	1
Karling et al., 2019	MES=0	35	88	63	0.67	0.68	23	12	36	17
Lee et al., 2019	MES=0	7	29	201	0.82	0.999	6	1	22	0
Ryu et al., 2019	MES=0	51	174	170	0.784	0.748	40	11	92	31
Ryu et al., 2019	UCEIS≤1	59	174	170	0.746	0.765	44	15	88	27
Hart et al., 2020	MES≤1	159	185	170	0.65	0.69	103	56	18	8
Bertani et al. 2020	MES≤1	46	97	157.5	0.75	0.889	35	11	45	6
Kim et al. 2020	MES=0	65	127	70	0.892	NA	58	7	44	18
Kim et al. 2020	MES≤1	98	127	200	0.888	NA	87	11	18	11
Yen et al. 2020	MES=0	15	50	156	0.8667	0.6286	13	2	22	13
Yen et al. 2020	MES≤1	31	50	156	0.7419	0.8421	23	8	16	3
Stevens et al. 2020 (wk 8)	MES≤1	509	595	251	0.77	0.69	392	117	59	27
Stevens et al. 2020 (wk 52)	MES≤1	304	351	99	0.63	0.81	192	112	38	9
Stevens et al. 2020 (wk 8)	MES=0	158	215	73	0.68	0.72	107	51	41	16
Stevens et al. 2020 (wk 52)	MES=0	172	198	76	0.73	0.62	126	46	16	10
Cannatelli et al. 2020	MES=0	29	76	112	0.897	0.851	26	3	40	7
Cannatelli et al. 2020	UCEIS≤1	33	76	148	0.935	0.822	31	2	35	8
Cannatelli et al. 2020	modPICaSSO≤3	33	76	161	0.879	0.767	29	4	33	10

Table S2. PRISMA 2009 Checklist. From: Moher D, Liberati A, Tetzlaff J, Altman DG, The PRISMA Group (2009). Preferred Reporting Items for Systematic Reviews and Meta-Analyses: The PRISMA Statement. PLoS Med 6(7): e1000097. doi:10.1371/journal.pmed1000097

Section/topic	#	Checklist item	Reported on page #
**TITLE**			
Title	1	Identify the report as a systematic review, meta-analysis, or both.	1
**ABSTRACT**			
Structured summary	2	Provide a structured summary including, as applicable: background; objectives; data sources; study eligibility criteria, participants, and interventions; study appraisal and synthesis methods; results; limitations; conclusions and implications of key findings; systematic review registration number.	1
**INTRODUCTION**			
Rationale	3	Describe the rationale for the review in the context of what is already known.	2
Objectives	4	Provide an explicit statement of questions being addressed with reference to participants, interventions, comparisons, outcomes, and study design (PICOS).	2
**METHODS**			
Protocol and registration	5	Indicate if a review protocol exists, if and where it can be accessed (e.g., Web address), and, if available, provide registration information including registration number.	2
Eligibility criteria	6	Specify study characteristics (e.g., PICOS, length of follow-up) and report characteristics (e.g., years considered, language, publication status) used as criteria for eligibility, giving rationale.	3
Information sources	7	Describe all information sources (e.g., databases with dates of coverage, contact with study authors to identify additional studies) in the search and date last searched.	3
Search	8	Present full electronic search strategy for at least one database, including any limits used, such that it could be repeated.	3
Study selection	9	State the process for selecting studies (i.e., screening, eligibility, included in systematic review, and, if applicable, included in the meta-analysis).	3
Data collection process	10	Describe method of data extraction from reports (e.g., piloted forms, independently, in duplicate) and any processes for obtaining and confirming data from investigators.	3
Data items	11	List and define all variables for which data were sought (e.g., PICOS, funding sources) and any assumptions and simplifications made.	3
Risk of bias in individual studies	12	Describe methods used for assessing risk of bias of individual studies (including specification of whether this was done at the study or outcome level), and how this information is to be used in any data synthesis.	3

Summary measures	13	State the principal summary measures (e.g., risk ratio, difference in means).	3
Synthesis of results	14	Describe the methods of handling data and combining results of studies, if done, including measures of consistency (e.g., I^2) for each meta-analysis.	3

Section/topic	#	Checklist item	Reported on page #
Risk of bias across studies	15	Specify any assessment of risk of bias that may affect the cumulative evidence (e.g., publication bias, selective reporting within studies).	3
Additional analyses	16	Describe methods of additional analyses (e.g., sensitivity or subgroup analyses, meta-regression), if done, indicating which were pre-specified.	3
**RESULTS**			
Study selection	17	Give numbers of studies screened, assessed for eligibility, and included in the review, with reasons for exclusions at each stage, ideally with a flow diagram.	3
Study characteristics	18	For each study, present characteristics for which data were extracted (e.g., study size, PICOS, follow-up period) and provide the citations.	3
Risk of bias within studies	19	Present data on risk of bias of each study and, if available, any outcome level assessment (see item 12).	3-9
Results of individual studies	20	For all outcomes considered (benefits or harms), present, for each study: (a) simple summary data for each intervention group (b) effect estimates and confidence intervals, ideally with a forest plot.	Tab.1, tab.2
Synthesis of results	21	Present the main results of the review. If meta-analyses are done, include for each, confidence intervals and measures of consistency	9
Risk of bias across studies	22	Present results of any assessment of risk of bias across studies (see Item 15).	9
Additional analysis	23	Give results of additional analyses, if done (e.g., sensitivity or subgroup analyses, meta-regression [see Item 16]).	
**DISCUSSION**			
Summary of evidence	24	Summarize the main findings including the strength of evidence for each main outcome; consider their relevance to key groups (e.g., healthcare providers, users, and policy makers).	9-11
Limitations	25	Discuss limitations at study and outcome level (e.g., risk of bias), and at review-level (e.g., incomplete retrieval of identified research, reporting bias).	11
Conclusions	26	Provide a general interpretation of the results in the context of other evidence, and implications for future research.	11
**FUNDING**			
Funding	27	Describe sources of funding for the systematic review and other support (e.g., supply of data); role of funders for the systematic review.	11

Figure S1. Flow diagram of data selection

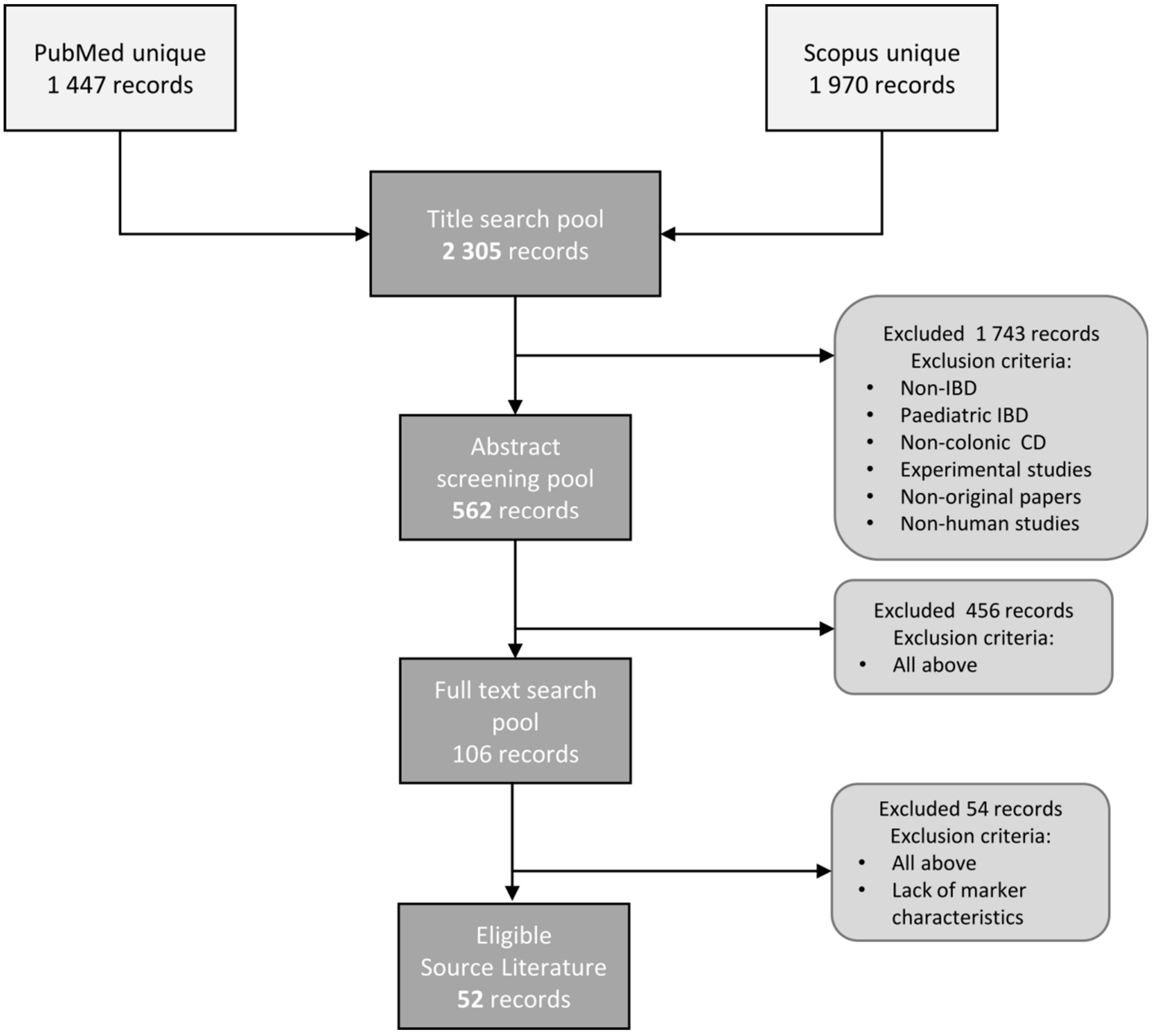

Supplement: Supplementary file 1 [file jcm-10-02203-s001.zip › jcm-1189114-supplementary.pdf]
